# The Optoelectronic, Vibrational, and Thermodynamic Attributes of 2D TMD-MoWX_4_ (X = S, Se) Alloys and Janus TMD-MoWS_2_Se_2_ Alloy: A DFT Approach

**DOI:** 10.1021/acsomega.5c06780

**Published:** 2025-10-15

**Authors:** Ally Siena Fernandes Gatinho, Caleb Nathan Navis, Leonardo de Souza Barbosa, Carlos Antonio Barboza, David Lima Azevedo, Edvan Moreira

**Affiliations:** † Postgraduate Program in Aerospace Engineering, State University of Maranhão (UEMA), Cidade Universitária Paulo VI, 65055-310 São Luís, Maranhão, Brazil; ‡ Department of Biophysics and Pharmacology, Federal University of Rio Grande do Norte (UFRN), Campus Universitário Lagoa Nova, 59072-970 Natal, Rio Grande do Norte, Brazil; § Institute of Physics, University of Brasilia (UnB), Campus Universitário Darcy Ribeiro—Asa Norte, Distrito Federal, 70919-970 Brasília, Brazil; ∥ Department of Physics, State University of Maranhão (UEMA), Cidade Universitária Paulo VI, 65055-310 São Luís, Maranhão, Brazil

## Abstract

This study presents a comprehensive theoretical investigation of two-dimensional transition metal dichalcogenide alloys, MoWS_4_, MoWSe_4_, and Janus MoWS_2_Se_2_—employing density functional theory and density functional perturbation theory. Phonon dispersion analysis confirms the dynamic stability in all monolayers. Electronic structure calculations demonstrate direct bandgaps along the Γ – *Y* high-symmetry path, with the hybrid HSE06 functional predicting significantly larger bandgaps (2.21, 1.95, and 2.08 eV) compared to GGA-PBE (1.68, 1.47, and 1.58 eV) and PBE + SOC (1.53, 1.33, and 1.43 eV) results for MoWS_4_, MoWSe_4_, and MoWS_2_Se_2_, respectively, emphasizing the importance of exchange–correlation treatments. Projected density of states (PDOS) analysis indicates predominant contributions from Mo/W d-orbitals near the Fermi level, while chalcogen p-orbitals substantially influence optical characteristics. Optical absorption spectra exhibit similar behaviors: MoWS_4_, MoWSe_4_, and MoWS_2_Se_2_ display strong absorption peaks near 5 and 10 eV, shown to be sensitive to the plane of polarization of the incident light in the range of ultraviolet radiation to the visible spectrum. Vibrational spectroscopy reveals unique fingerprints through prominent IR-active modes at 361 cm^–1^ (MoWS_4_), 280 cm^–1^ (MoWSe_4_), and 335 cm^–1^ (MoWS_2_Se_2_), complemented by Raman-active modes at 361 cm^–1^, 289 cm^–1^, and 335 cm^–1^, respectively. Negative free energies across 0–1000 K evidenced thermodynamic stability, while MoWSe_4_ exhibited superior stability. Molecular quantum dynamics simulations at 300 K further validate the thermal stability with minimal structural deformations. All these findings highlight the significant potential of MoWX_4_-based monolayers for advanced optoelectronic, thermoelectric, and sensing applications while establishing a robust theoretical framework to guide future experimental research and property optimization efforts.

## Graphical abstract

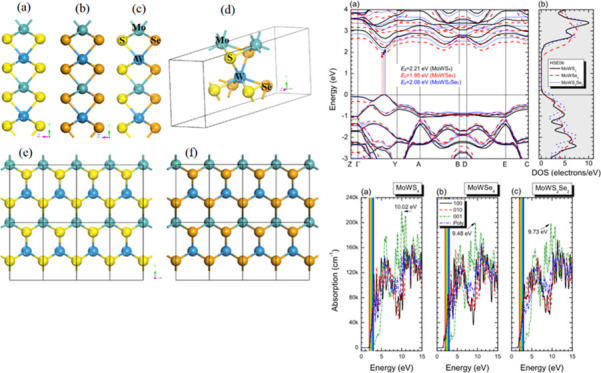

## 1Introduction

The synthesis of graphene has stimulated the development of new low-dimensional materials and placed two-dimensional (2D) materials at the frontier of materials science.^1^ Graphene excels at conducting electricity, but due to its lack of bandgap, its ability to modulate/switch electrical currents is limited.^2^ This fact has boosted the search for novel 2D semiconductor materials with transistor, optoelectronic, and spintronic applications. In recent years, transition metal dichalcogenides (TMDs) have emerged as a class of 2D materials with extraordinary properties that have attracted considerable attention.^3^ The most well-known and extensively investigated representative of this class is MoS_2_ (molybdenum disulfide). MoS_2_ can be found in different polymorphic phases such as hexagonal (2H-MoS_2_), rhombohedral (3R-MoS_2_), and tetragonal (1T-MoS_2_), where the 2H phase is more stable than the 3R and 1T phases.^4^ Bulk MoS_2_ is an indirect gap semiconductor (∼1.2 eV),^5^ while the MoS_2_ monolayer is a direct gap semiconductor (1.8–1.9 eV).^6,7^

MoS_2_ has made great breakthroughs in the fields of transistors^6,8^ and optoelectronic devices.^9,10^ Studies in the MoS_2_ monolayer optical properties indicated potential applications in magneto-optics and valleytronics.^6,11,12^ Furthermore, the MoS_2_ monolayer possesses higher photoluminescence (PL) intensity and anisotropic optical properties.^6,9,10,13^ For comparison, the PL intensity of the MoS_2_ monolayer is 10^4^ times higher than that of its bulk counterpart.^7^ Beyond that, MoS_2_ exhibits a relatively high absorption coefficient for wavelengths ranging from 400 to 500 nm,^7^ making it suitable for applications in solar cells. In addition, single-junction solar cells using TMD films as thin as 50 nm can reach up to 25% PCE, making them ideal for high-specific-power photovoltaics.^14,15^ There is a wide range of applications for MoS_2_, e.g., in biosensing and optical sensors and bioapplications such as DNA, cancer, and coronavirus (SARS-CoV-2) detection.^7,16–20^ In addition, MoS_2_ has a good adsorption capacity for heavy metal ions (e.g., Hg) and is being investigated to remove these pollutants from water.^21^ In addition to MoS_2_, WS_2_ (tungsten disulfide) is a promising material with outstanding electrical, magnetic, optical, and mechanical properties.^22^ Due to these properties, WS_2_ and WS_2_-related nanostructures have potential applications in optical and electrical devices, such as LEDs, sensors, field effect transistors (FETs), photodetectors, and memory devices.^22^ Other monolayers such as MXenes^23–26^ and transition metal dihalides (TMDHs)^27,28^ are promising materials for several technological applications.

In recent years, interest in Janus transition metal dichalcogenides (TMDs) has increased in different technological areas. The MXY Janus TMD monolayers (M = Mo, W; X, Y = S, Se, Te) were first investigated by Cheng et al.^29^ from a theoretical perspective.^30^ Finally, Janus TMD MoSSe was reported in 2017,^31^ revealing that an intrinsic electrostatic dipole exists in the direction perpendicular to the Janus MoSSe plane.^30–32^ Associated with this dipole perpendicular to the planar structure of Janus TMDs, these monolayers can greatly enhance the piezoelectric effect.^30^ Another interesting feature of Janus TMDs is that calculations predicted that symmetry breaking of Janus structures induces an electric field leading to new properties, such as the large Rashba effect and the formation of strongly correlated electronic states.^33^ These reported properties of Janus TMD make them candidates for applications in FETs, electrocatalysis, photocatalysis, spintronics, and piezoelectric devices.^30^ Beyond Janus MoSSe, Janus WSSe was also experimentally reported.^33,34^

Various TMD bulk alloys have been synthesized,^35^ e.g., Mo_1–*x*_W_*x*_S_2_,^36^ Mo_1–*x*_W_*x*_Se_2_,^37^ TaS_2(1–*x*)_Se_2*x*_,^38^ ZrS_2(1–*x*)_Se_2*x*_,^39^ HfS_2(1–*x*)_Se_2*x*_,^40^ and ReS_2(1–*x*)_Se_2*x*_,^41^ with most of these reported TMD bulk alloys capable of being mechanically exfoliated into monolayers.^35^

TMD alloy nanosheets have been studied for potential applications in electronics, optoelectronics, catalysis, and topological devices.^42^ Furthermore, TMD alloy monolayers also show interesting features such as tunable photoluminescence emissions, carrier-type modulation, and a high on/off ratio in FETs. Mo_1–*x*_W_*x*_S_2_ monolayers are the first exfoliated TMD monolayer alloy obtained.^43–45^ The syntheses of few-layer colloidal TMD alloy nanostructures Mo_*x*_W_1–*x*_Se_2_ and WS_2*y*_Se_2(1–*y*)_ were reported. These two alloys present gradually tunable optical properties and excitonic transitions that range from 1.51 to 1.93 eV.^42^ Other monolayers of TMD alloys, such as MoS_2(1–*x*)_Se_2*x*_,^46–50^ WS_2(1–*x*)_Se_2*x*_,^51^ and Mo_1–*x*_W_*x*_Se_2_ ^44,52^ have been experimentally reported. Recently, the (MoWV)­Se_2_ ternary alloy nanosheet was synthesized by colloidal reaction, where the ternary alloy has the potential to alter the electronic structure of the materials to make them suitable for electrochemical applications.^53^

The applications of 2D TMD alloys are varied. For example, field effect transistors (FETs) with high on/off ratios are expected for semiconducting monolayer TMD alloys.^35^ Photodetecting devices based on MoS_2_ monolayers have already demonstrated a high sensitivity of up to 800 A W^–1^, with a response of a few seconds,^54^ and it is expected that TMD monolayer alloys have similar potential for high photodetecting sensitivity.^35^ Furthermore, photon detection using MoS_2(1–*x*)_Se_2*x*_ monolayers revealed photocurrent generation with increasing illumination power.^35,55^ Moreover, 2D TMD alloys have applications in hydrogen evolution reaction (HER), with MoS_2(1–*x*)_Se_2*x*_ showing a better HER performance.^35^

As presented above, significant progress has been made in the synthesis and characterization of 2D and Janus TMDs in recent years. However, both experimental and first-principles investigations of TMD alloy monolayers are relatively lacking compared with conventional 2D TMDs. First-principles calculations are important in predicting the stability, electronic structure, and optical response of new TMD alloys, which guide and inspire future experiments. Therefore, computational investigation of 2D TMD alloys can provide valuable information about their potential for optoelectronic applications.

Based on the theoretical results of the 2D TMD, Janus TMD, and 2D TMD alloys, the structural, electronic, optical, vibrational, and thermodynamic characteristics of the two-dimensional TMD-MoWX_4_ (X = S, Se) and Janus TMD-MoWS_2_Se_2_ alloys were investigated using density functional theory (DFT). In addition, cohesive energies, quantum dynamics, and IR and Raman spectra were calculated and assigned. This work is organized as follows. Section 2 presents the computational method. Section 3 discusses the results and discussion regarding the physical properties of the 2D TMD-MoWX_4_ (X = S, Se) alloys and Janus TMD-MoWS_2_Se_2_ alloy, including the structural details, geometry optimization in the ground state, and electronic properties, including the band structure and density of states (DOS). In addition, the dielectric function, optical absorption, phonon dispersion, infrared and Raman spectra, thermodynamic properties, and quantum dynamics are described in Section 3. Conclusions are presented in Section 4.

## 2Computational Method

The structural, electronic, optical, vibrational, and thermodynamic properties of the MoWS_4_, MoWSe_4_, and Janus MoWS_2_Se_2_ monolayers were investigated using first-principles calculations based on Density Functional Theory (DFT), within the Kohn–Sham framework^56,57^ and the plane wave pseudopotential method^58^ implemented using Cambridge Sequential Total Energy Package (CASTEP) code^59,60^ to obtain the lowest total energy state of the primitive cell. Exchange–correlation was treated using the Generalized Gradient Approximation (GGA) with the Perdew–Burke–Ernzerhof (PBE) parametrization^61,62^ to obtain the cohesive energy, structural, optoelectronic, vibrational, and thermodynamic properties. Spin–orbit coupling (SOC) effects, calculated using the PBE method, were also considered to evaluate the band structures of the monolayers. To improve the precision of the electronic bandgap, single-point calculations were also performed using the hybrid Heyd–Scuseria–Ernzerhof functional (HSE06).^63^ The Brillouin zone (BZ) was sampled using a 3 × 4 × 1 Monkhorst–Pack *k*-point mesh,^64^ with electronic structure calculations incorporating the valence electron configurations specific to each constituent element: Mo-4d^5^5s^1^, W-5d^4^6s^2^, S-3s^2^3p^4^, and Se-4s^2^4p^4^. The vacuum distance applied along the out-of-plane direction to avoid interactions between periodic images is set to at least 18.0 Å.^2,28^

To obtain accurate structural optimization, a plane-wave basis set was limited by a cutoff energy of 720 eV, considering the following convergence thresholds: variations in total energy less than 0.5 × 10^–5^ eV per atom, maximum ionic forces under 0.01 eV/Å, residual stress below 0.02 GPa, and atomic displacements not exceeding 0.5 × 10^–3^ Å. Atomic relaxations were performed using the Broyden–Fletcher–Goldfarb–Shanno (BFGS) algorithm,^65^ essential to ensure proper convergence toward the system’s ground-state energy. As part of the BFGS algorithmic procedure, for each self-consistent field step, the electronic minimization parameters were atomic convergence tolerance for the total energy per atom of 0.5 × 10^–6^ eV, eigenenergy convergence tolerance of 0.1667 × 10^–6^ eV, and a convergence window of 3 cycles. The vibrational behavior of the systems was investigated using density functional perturbation theory (DFPT), based on the linear response formalism.^66,67^ To verify the thermal stability of the TMD and TMD Janus alloys, quantum dynamics simulations were performed, using DMol3 software,^68,69^ at 300 K within the *NVT* ensemble with the Nosé-Hoover thermostat,^70–72^ using a supercell of 2 × 2 × 1, considering a 1 × 2 × 1 *k*-point grid, with a total duration of 5 ps and a time step of 1 fs. The ab initio quantum dynamics was performed using LDA-PWC^73–75^ and the DNP (double numerical plus polarization) basis set.

The formation energies were calculated using QuantumATK W-2024.09 software^76–79^ and GGA-PBE. The optimization geometry parameters used were a force tolerance of 0.01 eV Å^–1^, a stress error tolerance of 0.02 GPa, a maximum step size of 0.2 Å, and the LBFGS (limited-memory BFGS) optimizer.^80^ A 4 × 4 × 4 *k*-point grid was used in all formation energy calculations and a medium basis set with PseudoDojo pseudopotential (optimized norm-conserving Vanderbilt—ONCV)^78^ with a cutoff energy of 13 Ha.

## 3Results and Discussion

### 3.1Geometry Optimization

The lattice parameters of the TMD and Janus TMD alloy monolayers (Figure 1) were calculated using DFT with the GGA-PBE functional and single-point calculations with the HSE06 hybrid functional, based on optimized geometries obtained by GGA-PBE. The calculations were performed using structural data obtained from the Computational 2D Materials Database (C2DB),^81,82^ with MoWS_4_ and MoWSe_4_ used as prototype structures.

**Figure 1:**
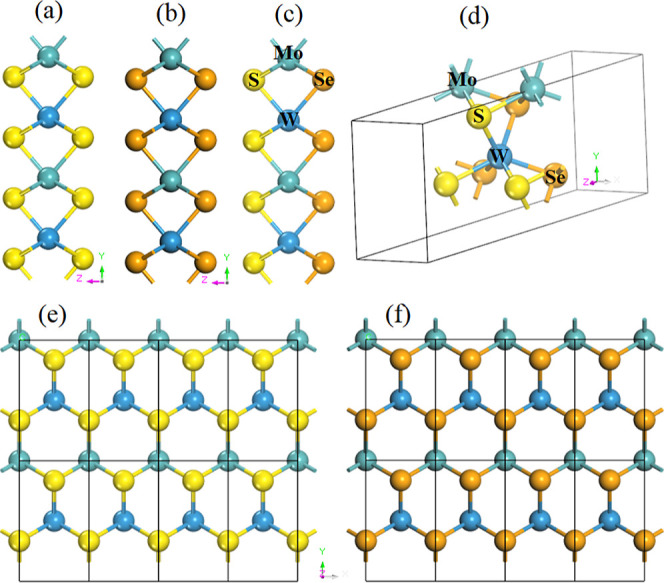
Side views of the atomic structure of (a) MoWS_4_, (b) MoWSe_4_, (c) MoWS_2_Se_2_; (d) crystal structure of monoclinic MoWS_2_Se_2_; top views of (e) MoWS_4_, and (f) MoWSe_4_ replicated in the *X* and *Y* directions. The honeycomb structure consists of Mo (green ball), W (blue ball), S (yellow ball), and Se (orange ball). (For interpretation of the references to colors in this figure legend, the reader is referred to the web version of this paper.).

The atomic structure of the MoWS_4_, MoWSe_4_, and MoWS_2_Se_2_ monolayers is displayed in Figure 1 from different perspectives, where light-green spheres represent Molybdenum (Mo) atoms, blue spheres represent Tungsten (W) atoms, yellow spheres represent Sulfur (S) atoms, and orange spheres represent selenium (Se) atoms. Figure 1 shows the side and top views of the 4 × 2 supercell of MoWS_4_ and MoWSe_4_ monolayers and the primitive cell of MoWS_2_Se_2_ (d), which has a *P*_1_ *m*_1_ space group of monoclinic symmetry, with a structure sandwiched between layers of chalcogen atoms from a side view (*a*, *b*, and *c*) and a honeycomb structure from a top view (*e* and *f*).

Table 1 presents the lattice parameters for these monolayers obtained from the generalized gradient approximation (GGA) with the Perdew–Burke–Ernzerhof (PBE) functional since the lattice parameters can be significantly closer to possible experimental results. After fully geometric optimization, the lattice constants for the MoWS_4_, MoWSe_4_, and MoWS_2_Se_2_ monolayers are as follows, respectively: *a* = 5.533 Å, 5.737 Å, and 5.633 Å, *b* = 3.197 Å, 3.186 Å, and 3.256 Å, and *c* = 18.193 Å, 18.134 Å, and 18.559 Å, with respective vertical separations following the literature for recent studies in the search for new materials using this methodology.^2,28^

**Table 1:** Lattice Parameters of the MoWS_4_, MoWSe_4_, and MoWS_2_Se_2_ ^a^

system	*a*	*b*	*c*	α = γ	β	*V*
MoWS_4_	5.533	3.197	18.193	90.0	90.000	321.873
MoWSe_4_	5.737	3.316	18.963	90.0	89.999	360.874
MoWS_2_Se_2_	5.633	3.256	18.559	90.0	90.014	340.533

a Lengths (*a*, *b*, and *c*) are in Å, angles (α, β, γ) in degrees (°), and volumes (*V*) in Å^3^.

For comparison, the calculated PBE lattice parameters (*a* = *b*) for conventional bulk TMDs are 3.194, 3.338, 3.194, and 3.312 Å for MoS_2_, MoSe_2_, WS_2_, and WSe_2_, respectively.^83^ The experimental values of the lattice constants of the bulk TMDs are 3.160, 3.288, 3.153, and 3.280 Å for MoS_2_, MoSe_2_, WS_2_, and WSe_2_, respectively.^83–85^ The calculated PBE lattice constants (*a* = *b*) for conventional 2D TMDs are 3.18, 3.32, 3.19, and 3.32 Å for 2H-MoS_2_, 2H-MoSe_2_, 2H-WS_2_, and 2H-WSe_2_, respectively.^86^ Finally, the experimental values for 2D TMDs are 3.15 ± 0.01, 3.27 ± 0.01, 3.2 ± 0.1, and 3.2757 ± 0.0008 Å for MoS_2_, MoSe_2_, WS_2_, and WSe_2_, respectively.^87–89^

Fractionary atomic coordinates for the monoclinic TMD-MoWX_4_ (X = S, Se) alloys and Janus TMD-MoWS_2_Se_2_ alloy were determined using the GGA-PBE functional, and these are shown in Tables 2, 3, and 4, respectively.

**Table 2:** Internal Atomic Coordinates for Monoclinic MoWS_4_ ^a^

MoWS_4_	internal coordinates		
element	*u*	*v*	*w*
S_1_	0.662	0.000	0.414
S_2_	0.662	0.000	0.586
S_3_	0.169	0.500	0.586
S_4_	0.169	0.500	0.413
Mo	0.001	0.000	0.499
W	0.497	0.500	0.499

a The coordinates (*u*, *v*, and *w*) are measured relative to the *a*, *b*, and *c* lattice parameters of the primitive cell, respectively.

**Table 3:** Internal Atomic Coordinates for Monoclinic MoWSe_4_ ^a^

MoWSe_4_	internal coordinates		
element	*u*	*v*	*w*
Se_1_	0.663	0.000	0.475
Se_2_	0.662	0.000	0.524
Se_3_	0.167	0.500	0.524
Se_4_	0.168	0.500	0.475
Mo	0.000	0.000	0.500
W	0.498	0.500	0.499

a The coordinates (u, v, and w) are measured relative to the a, b, and c lattice parameters of the primitive cell, respectively.

**Table 4:** Internal Atomic Coordinates for Monoclinic MoWS_2_Se_2_ ^a^

MoWS_2_Se_2_	internal coordinates		
element	*u*	*v*	*w*
S_1_	0.662	0.000	0.585
S_2_	0.169	0.500	0.586
Se_1_	0.663	0.000	0.411
Se_2_	0.168	0.500	0.411
Mo	0.000	0.000	0.502
W	0.497	0.500	0.502

a The coordinates (*u*, *v*, and *w*) are measured relative to the *a*, *b*, and *c* lattice parameters of the primitive cell, respectively.

### 3.2Cohesive Energy and Formation Energy

The cohesive energy indicates the strength with which the atoms are bonded within a crystal structure, reflecting the stability and integrity of the nanomaterials.^26,90^ The formal definition of cohesive energy, *E*_coh_, is the difference between the total energy of the system and the sum of the energy of the individual atoms divided by the number of atoms in each system. For the TMD and Janus alloys, the cohesive energy is as follows^15^

1 $$E_{coh}=\frac{E_{alloy}-\sum E_{isolated}}{N}$$

where *E*_isolated_ are the energies of the isolated Mo, W, S, and Se atoms, respectively. The *E*_alloy_ is the total energy of the unit cell of each 2D TMD-MoWX_4_ (X = S, Se) alloy and Janus TMD-MoWS_2_Se_2_ alloy with *N* number of atoms. The number of atoms in each unit cell is *N* = 6. The calculation of the cohesive energies followed the same convergence criteria detailed in Section 2.

The moduli of the cohesive energy obtained for MoWS_4_, MoWSe_4_, and MoWS_2_Se_2_ are 5.167, 4.770, and 4.798 eV/atom, respectively. Thus, the alloy with the highest cohesive energy is MoWS_4_ (5.167 eV/atom). This result shows that all of the TMD alloys studied have higher cohesive energy, indicating that the proposed monolayers present energetic stability. The results obtained are close to the cohesive energies of the MoS_2_ bulk (4.960 eV/atom) and monolayer (4.979 eV/atom).^91^ The modulus of the cohesive energies per atom of all TMD alloys are higher than CrS_2_ (4.10 eV/atom^92^ and 4.08 eV/atom^93^), CrSe_2_ (3.59 eV/atom^92^) and CrSSe (3.83 eV/atom^92^), calculated with a similar methodology used here. The modulus of the cohesive energy obtained for the TMD and TMD Janus alloys is also higher than other monolayers such as phosphorene (3.48 eV/atom) and silicene (3.96 eV/atom).^94^

The formation energies (*E*_form_) were also calculated for all of the TMD alloys. These calculations were performed using QuantumATK W-2024.09 software.^76–79^ To obtain these formation energies, the total energies of the most stable periodic reference structures of the constituent elements (Mo, W, S, and Se) were calculated, and the corresponding atomic energies were taken as the chemical potentials. For Mo, the three-dimensional body-centered cubic phase (bcc) with the space group *Im*3̅*m* was considered. For W, the reference was also bcc with space group *Im*3̅*m*. For S, the monoclinic S_32_ phase with the space group *P*2/*c* was employed. Finally, for Se, the hexagonal phase with the space group *P*3_1_21 was used. The formation energy values above were then calculated using the following equation

2 $$E_{form}=E_{alloy}-\sum_{i}n_{i}\mu_{i}$$

where, *n*_*i*_ is the number of atoms of the element in the alloy and μ_*i*_ is the chemical potential of the element. The values obtained for the formation energies of each TMD alloy were −5.253 eV, −3.240 eV, and −4.170 eV for MoWS_4_, MoWSe_4_, and MoWS_2_Se_2_, respectively. For comparison, the formation energy of the MoS_2_ monolayer is −2.70 eV in GGA-PBE.^95^ The fact that the formation energies of the TMD alloys are more negative than those of MoS_2_ at the same level of calculations indicates that these TMD alloys are thermodynamically more stable than the isolated MoS_2_ monolayer.

### 3.3Electronic Properties

Figures 2–4, and 5 show the Kohn–Sham electronic band structures, which describe the electronic energy dispersion *E*(*k*) in the first Brillouin Zone (BZ), along with the projected density of states (PDOS) for the MoWS_4_, MoWSe_4_, and MoWS_2_Se_2_ monolayers, calculated using both the GGA-PBE and HSE06 methods (atom-projected contributions will be detailed separately). In all cases, the Fermi level (denoted by a dashed black line) is set at 0 eV. The DFT calculations used high-symmetry BZ paths (Figure 6) following the sequence: *Z*(0,0,0.5) → Γ­(0,0,0) → *Y*(0.5,0,0) → *A*(0.5,0.5,0) → *B*(0,0,0.5) → *D*(0,0.5,0.5) → *E*(0.5,0.5,0.5) → *C*(0.5,0,0.5), with reciprocal lattice vectors g_1_, g_2_, and g_3_ shown for reference. The GGA-PBE calculations predict direct bandgaps of 1.68 eV (MoWS_4_), 1.47 eV (MoWSe_4_), and 1.58 eV (MoWS_2_Se_2_), while HSE06 yields larger values of 2.21, 1.95, and 2.08 eV, respectively, with the maximum valence band fixed at 0 eV. The bandgap value of MoWS_2_Se_2_ calculated with HSE06 is comparable to the HSE06 bandgap reported for Janus MoSSe (2.09 eV).^96^ A comparison of the PBE and PBE + SOC electronic band structures, depicted in Figures 2–4, reveals that the inclusion of SOC influences the electronic bandgap of the three TMD alloys. In all cases, the bandgap decreases when SOC is taken into account. The PBE + SOC calculations predict direct bandgaps of 1.53 eV (MoWS_4_), 1.33 eV (MoWSe_4_), and 1.43 eV (MoWS_2_Se_2_), respectively. Furthermore, SOC leads to band splitting at the Γ → Y high-symmetry point within the conduction and valence states in all three studied monolayer structures. Experimental bandgaps of 2D MoS_2_ and 2D MoSe_2_ are 1.86 eV^97^ and 1.55 eV,^98^ respectively. The experimental bandgaps of 2D WS_2_ and 2D WSe_2_ are 1.98–2.05 eV^99,100^ and 1.62–1.66 eV,^100^ respectively. Beyond that, the experimental bandgap value of the Janus MoSSe monolayer is 1.68 eV.^31,96^ These experimental bandgap values are comparable to those of the TMD alloys studied here.

**Figure 2:**
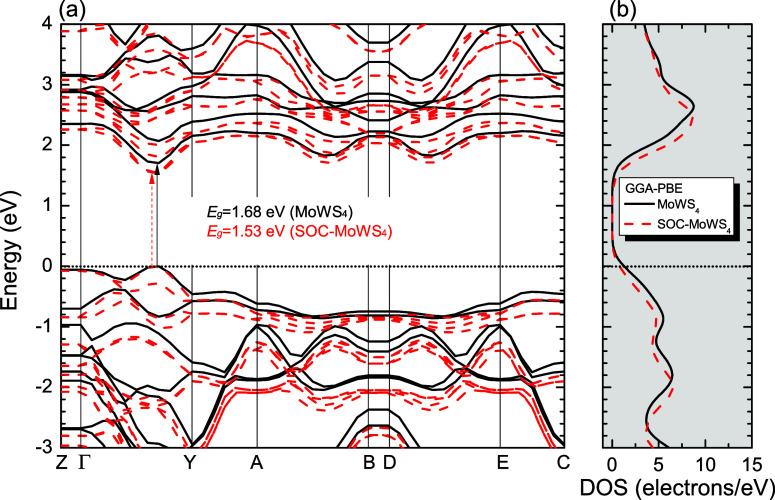
(a) Band structures of MoWS_4_ (solid black lines) and SOC band structures of MoWS_4_ (dashed red lines). (b) Total density of states (DOS) calculated by using the GGA-PBE approximation. The Fermi level is indicated as the line at *E* = 0 eV (for interpretation of the references to colors in this figure legend, the reader is referred to the web version of this paper).

**3:**
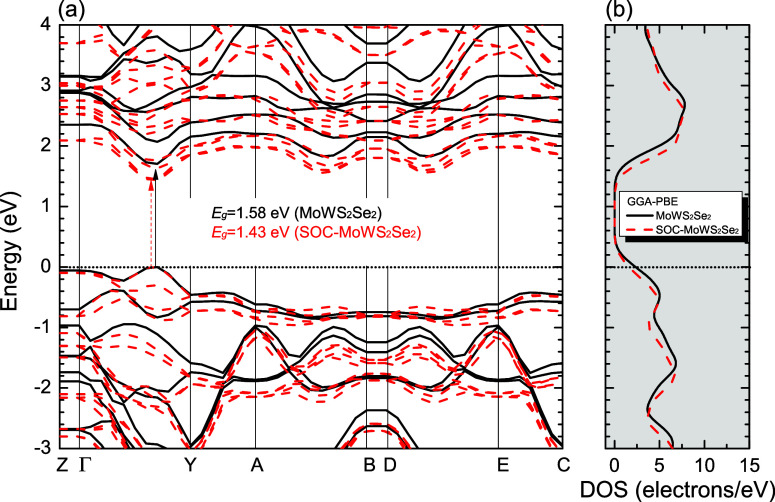
(a) Band structures of MoWS_2_Se_2_ (solid black lines), and SOC MoWS_2_Se_2_ (dashed red lines). (b) Total density of states (DOS) calculated by using the GGA-PBE approximation. The Fermi level is indicated as the line at *E* = 0 eV (for interpretation of the references to colors in this figure legend, the reader is referred to the web version of this paper).

**Figure 4:**
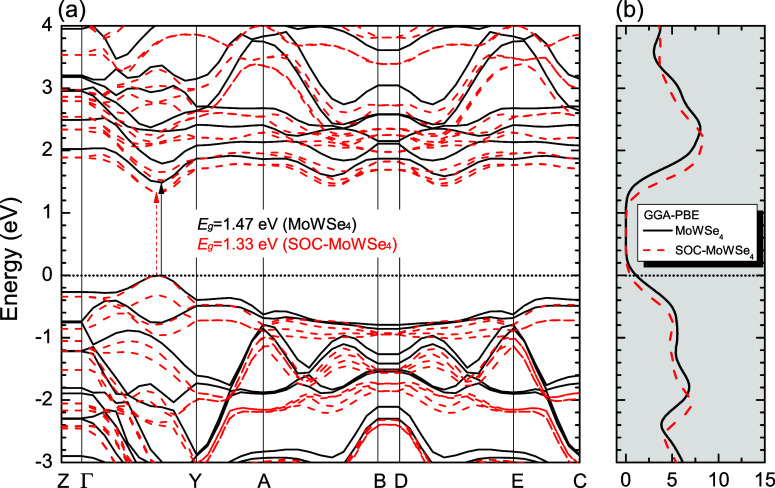
(a) Band structures of MoWSe_4_ (solid black lines) and SOC band structures of MoWSe_4_ (dashed red lines). (b) Total density of states (DOS) calculated by using the GGA-PBE approximation. The Fermi level is indicated as the line at *E* = 0 eV (for interpretation of the references to colors in this figure legend, the reader is referred to the web version of this paper).

**Figure 5:**
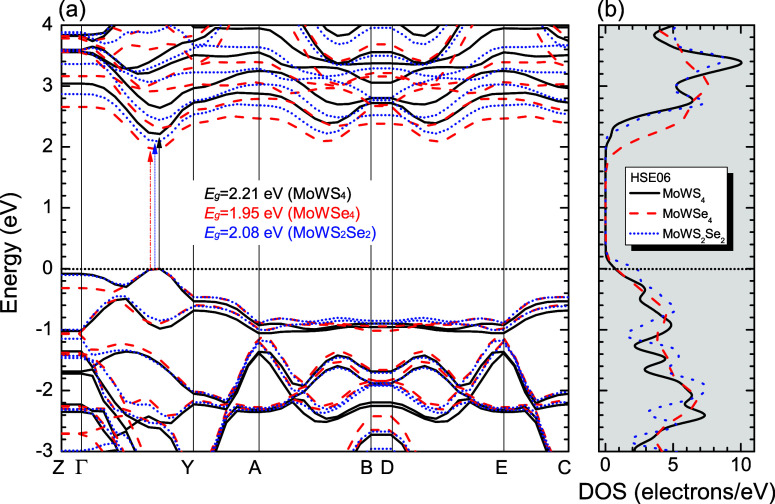
(a) Band structures of MoWS_4_ (solid black lines), MoWSe_4_ (dashed red lines) and MoWS_2_Se_2_ (dotted blue lines). (b) Total density of states (DOS) calculated using the HSE06 hybrid functional. The Fermi level is indicated as the line at *E* = 0 eV (for interpretation of the references to colors in this figure legend, the reader is referred to the web version of this paper).

**Figure 6:**
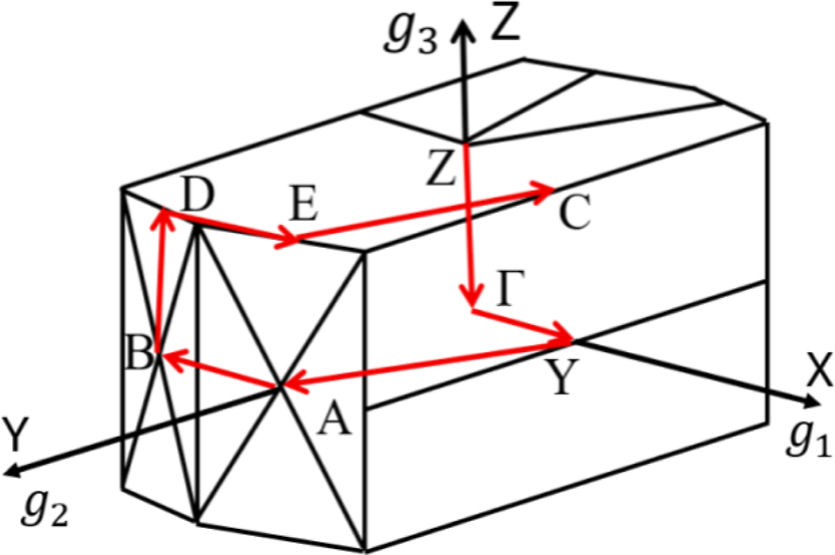
First Brillouin zone of the monolayers, showing high-symmetry points and the primitive vectors of their reciprocal lattices.

The observed variation in bandgaps is not solely attributable to SOC. Notably, materials containing sulfur (S) tend to exhibit larger bandgaps, an effect driven by the higher electronegativity of this element, which results in higher binding energies and, consequently, a wider separation between the valence and conduction bands. Conversely, Selenium (Se), being less electronegative and possessing a larger atomic radius, attracts electrons less strongly, leading to a bandgap reduction, as evidenced by the lower values of MoWSe_4_.

Beyond these chemical factors, symmetry breaking in the MoWS_2_Se_2_ alloy plays a crucial role. Its Janus structure, terminated by a face of S atoms and another face of Se atoms, generates an intrinsic electric dipole perpendicular to the plane. This dipole modifies the electrostatic potential and distorts the crystal lattice, resulting in a bandgap value intermediate between those of MoWS_4_ and MoWSe_4_, which accurately reflects the hybrid and asymmetric nature of this material.

PDOS analysis (Figures 7 and 8) reveals similar orbital contributions across all monolayers: Mo-4d^5^/5s^1^, W-4f^1^/5d^4^/6s^2^, S-3s^2^/3p^4^, and Se-3d^1^/4s^2^/4p^4^. Throughout the −3.0 to 5.0 eV range, Mo-d^5^ and W-d^4^ orbitals dominate, while chalcogen contributions differentiate the systems: Se-p^4^ prevails in MoWSe_4_, S-p^4^ in MoWS_4_, and both contribute comparably in MoWS_2_Se_2_, maintaining this trend above and below the Fermi level.

**Figure 7:**
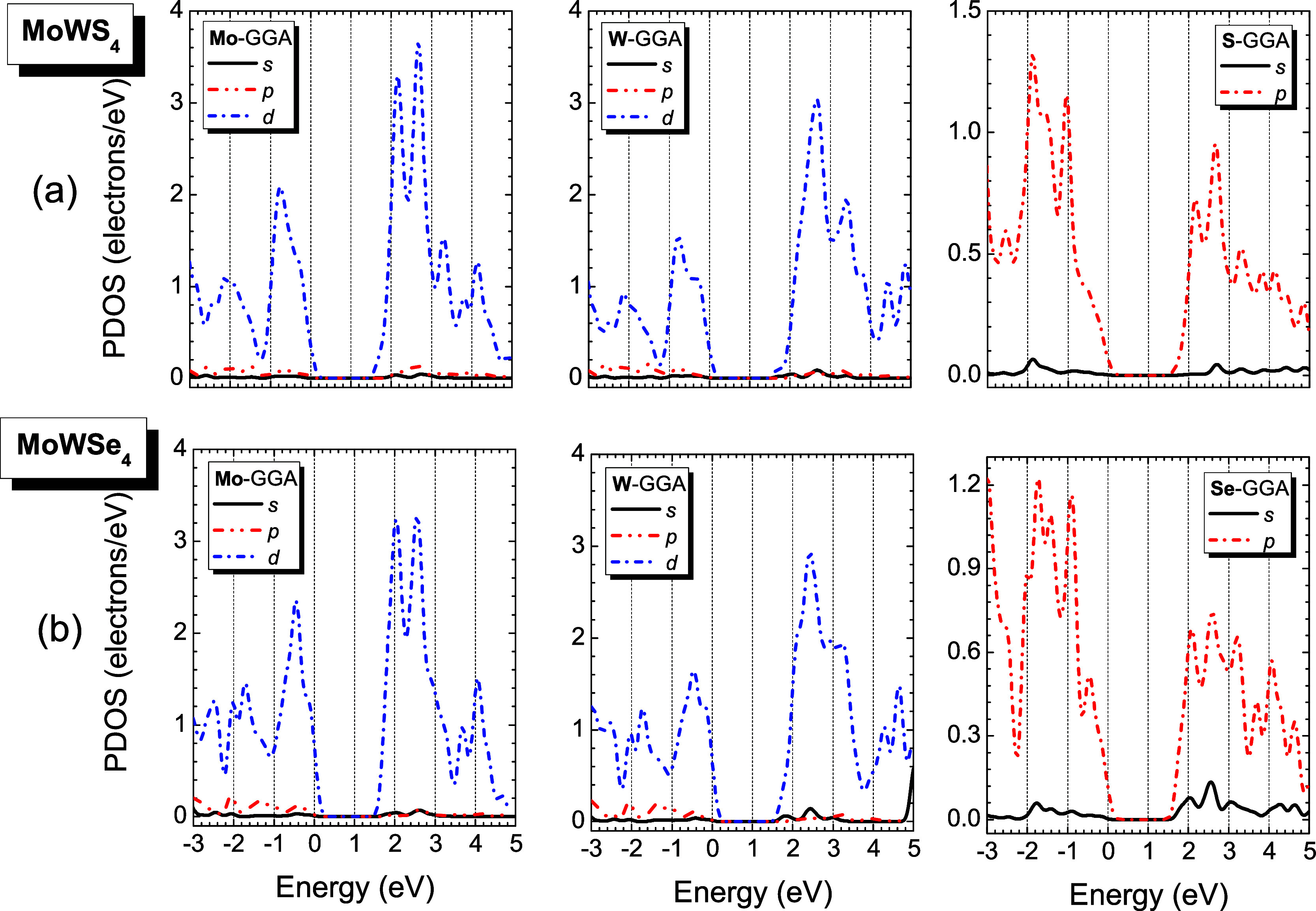
GGA-PBE partial density of states (PDOS) of monoclinic (a) MoWS_4_ and (b) MoWSe_4_ per atom of molybdenum (Mo), tungsten (W), sulfur (S), or selenium (Se), and per orbital type (s-solid black line, p-dashed red line, and d-dotted blue line). The Fermi level is indicated as the line at *E* = 0 eV (for interpretation of the references to colors in this figure legend, the reader is referred to the web version of this paper).

**Figure 8:**
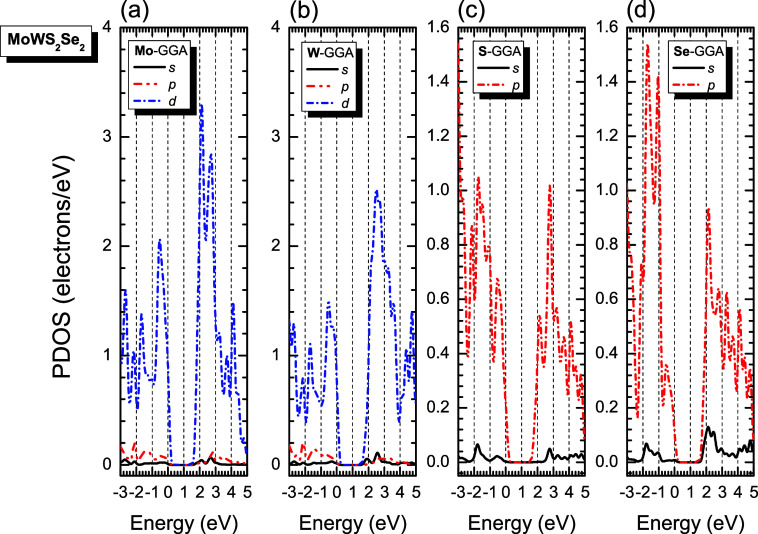
GGA-PBE partial DOS (PDOS) of monoclinic MoWS_2_Se_2_ per atom (a) molybdenum, (b) tungsten, (c) sulfur, (d) selenium, and per orbital type (s-solid black line, p-dashed red line, and d-dotted blue line). The Fermi level is indicated as the line at *E* = 0 eV (for interpretation of the references to colors in this figure legend, the reader is referred to the web version of this paper).

### 3.4Optical Properties

The optical properties of the monolayers were investigated, obtaining the real part of the dielectric function from its imaginary component through the Kramers–Kronig relations.^101,102^ The results show remarkable consistency across all three monolayer systems (MoWS_4_, MoWSe_4_, and MoWS_2_Se_2_). Figures 9 and 10 show both the real and imaginary components of ϵ­(ω) for these structures, with calculations performed for polarized light in three crystallographic orientations ([100], [010], and [001]) as well as for polycrystalline samples (POLY), with an emphasis on the energy values of the most pronounced peaks. The analysis indicates minimal optical anisotropy for the [100] and [010] polarizations, with slightly more pronounced (though still modest) anisotropy observed for the [001] direction and polycrystalline case. More importantly, all three monolayer materials exhibit similar behavior patterns in their optical response.

**Figure 9:**
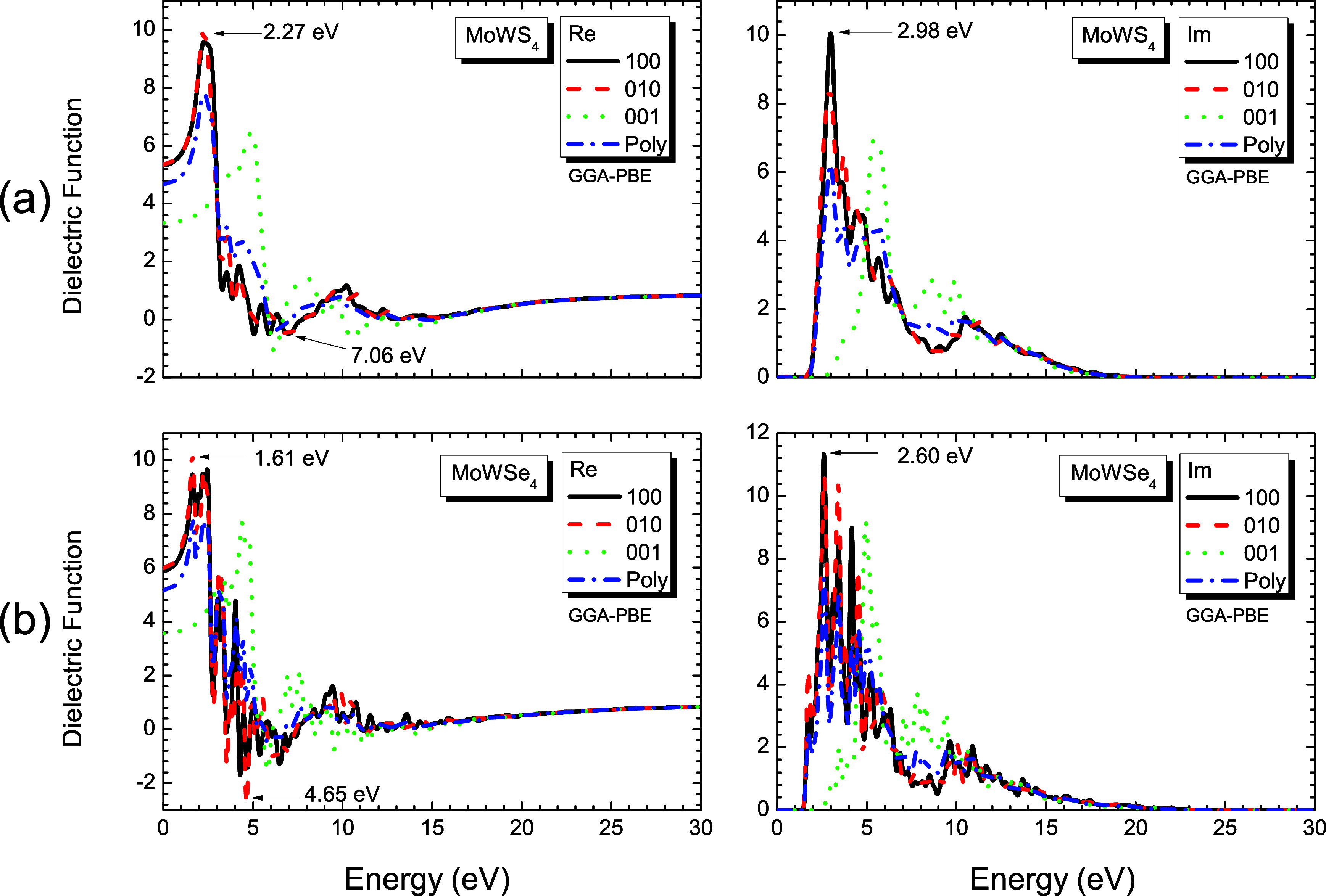
Real (Re) and imaginary (Im) parts of the dielectric response functions as a function of energy (eV) for (a) MoWS_4_ and (b) MoWSe_4_ monolayer structures, along the directions [100], [010], [001], and for a polycrystalline sample (Poly).

**Figure 10:**
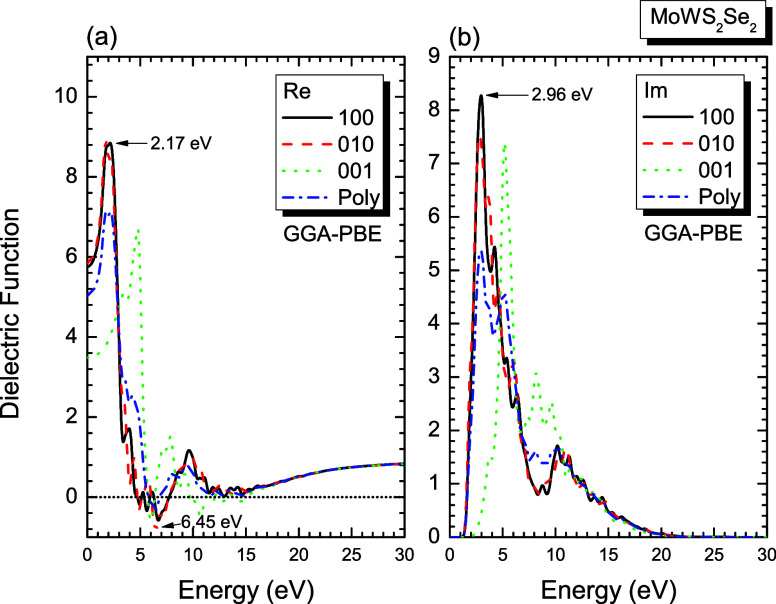
(a) Real (Re) and (b) imaginary (Im) parts of the dielectric response functions as a function of energy (eV) for MoWS_2_Se_2_ monolayer structures along the directions [100], [010], [001], and for a polycrystalline sample (Poly).

The absorption coefficient measures the energy lost when an electromagnetic wave passes through a material, considering that its intensity decays exponentially with the distance from the surface of incidence. Figure 11 shows the optical absorption spectra calculated for the three monolayers. MoWS_4_, MoWSe_4_, and MoWS_2_Se_2_ monolayers exhibit similar behavior, with the strongest absorption peaks along the [001] direction at 10.02, 9.48, and 9.73 eV, respectively, associated with electronic transitions between the highest valence band (dominated by Mo-d^5^ and W-d^4^ orbitals) and the conduction bands derived from S-p^4^ and Se-p^4^ orbitals. The differences in absorption can be attributed to several factors: (a) Selenium (Se) is a heavier element with a larger atomic radius than sulfur (S), which reduces the bandgap energy between the valence and conduction bands in MoWSe_4_ compared to MoWS_4_ (see bandgap values in Figures 4 and 5), (b) smaller bandgaps shift optical absorption to lower energies, potentially reducing absorption in the visible region, (c) the charge polarizability of Se is higher than that of S, strengthening electron–electron interactions and possibly weakening direct optical excitations, thereby decreasing absorption in certain energy ranges. Lastly, replacing S with Se modifies the hybridization of the metal (d orbitals of Mo/W) and chalcogen (p orbitals) states, altering the electronic density of states near the bandgap (Figure 7).^46^

**Figure 11:**
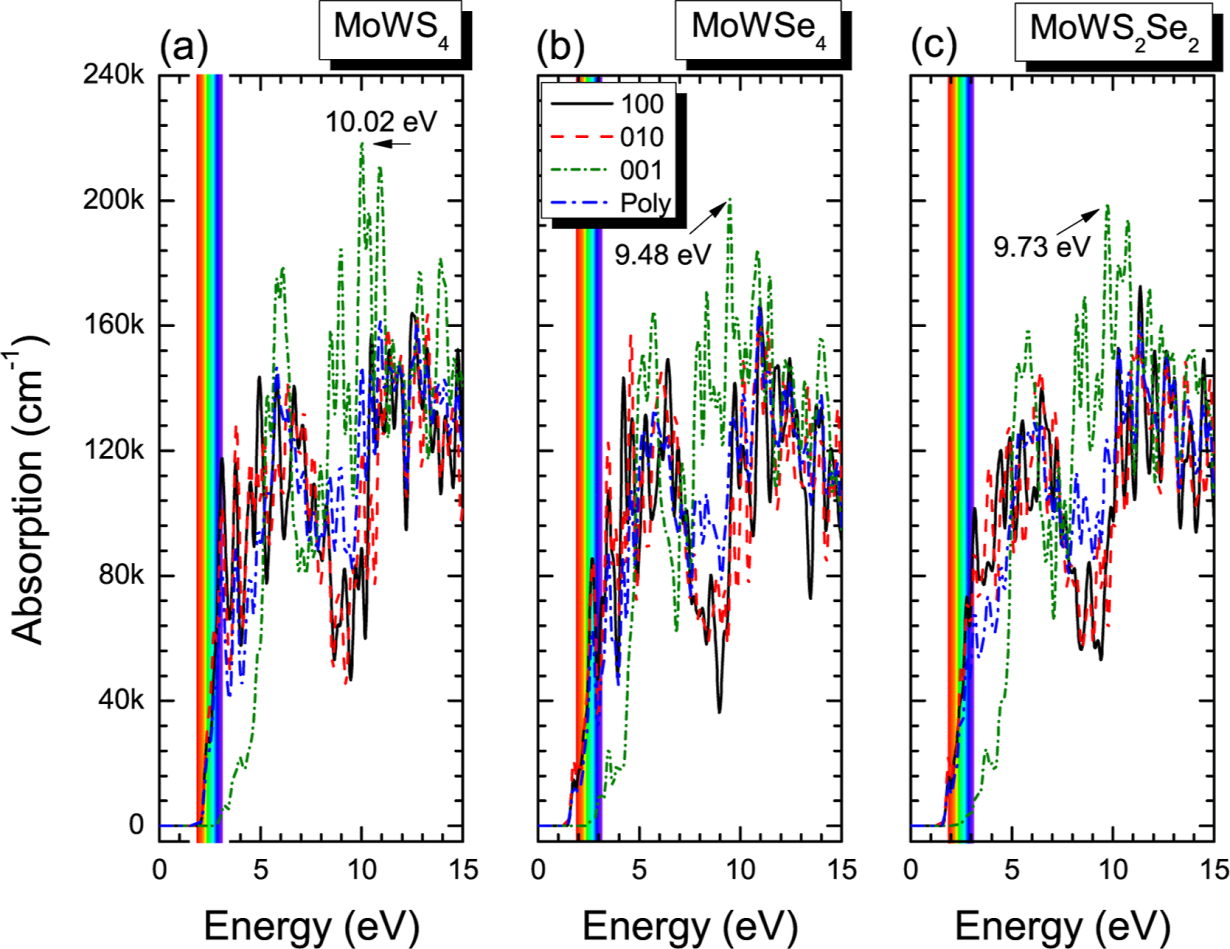
Optical absorption spectra of (a) MoWS_4_, (b) MoWSe_4_, and (c) MoWS_2_Se_2_, when the incident radiation is polarized along the crystalline planes [100], [010], [001], and for a polycrystalline sample (Poly).

### 3.5Phonon Dispersion Curves

Figure 12 shows the phonon dispersion curves for MoWS_4_ (black lines), MoWSe_4_ (dashed red lines), and MoWS_2_Se_2_ (dashed blue lines) within the frequency range 0 to 500 cm^–1^. Phonon dispersion dictates the dynamic behavior and thermal properties of the materials. The phonon dispersion curves show no imaginary frequencies, suggesting that MoWS_4_, MoWSe_4_, and MoWS_2_Se_2_ are at the local minimum since all phonon frequencies are positive throughout the Brillouin zone, which would indicate a possible monolayer stability. Figure 12 also reveals that all three monolayers have 18 vibrational modes, three of which are acoustic contributions corresponding to the 0 to 147 cm^–1^ (maximum value for MoWS_4_) region. When the dispersion curves of Figure 12a–c are compared, a difference is observed in the configuration of the optical modes.

**Figure 12:**
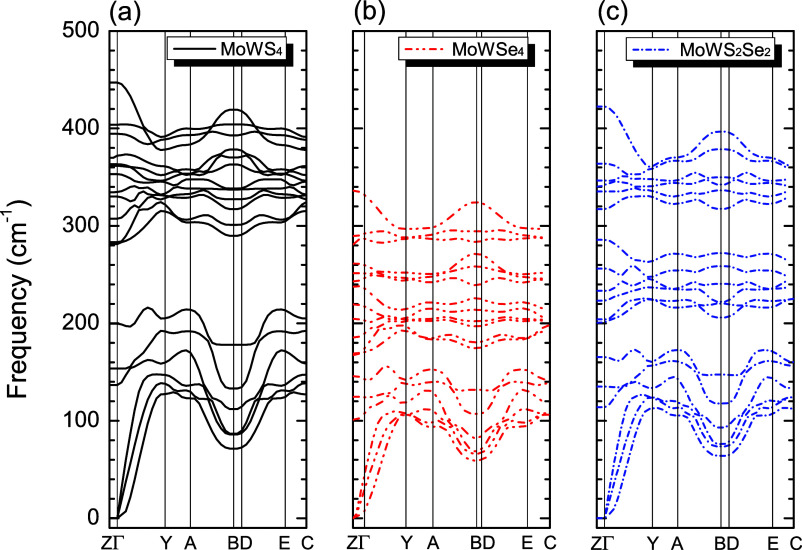
Phonon dispersion of the (a) MoWS_4_ (black lines), (b) MoWSe_4_ (red dashed lines), and (c) MoWS_2_Se_2_ (blue dashed lines) structure for frequencies ranging from 0 to 500 cm^–1^ (for interpretation of the references to colors in this figure legend, the reader is referred to the web version of this paper).

The spectrum for the MoWS_4_ structure (Figure 12a) shows higher frequencies, especially in the optical modes. This indicates stronger atomic bonds, which is attributed to the predominant presence of lighter atoms, such as sulfur (S). The optical branches are well separated from the acoustic branches, suggesting good dynamic stability and less coupling between the acoustic and optical vibrations. For MoWSe_4_ (Figure 12b), it is observable that replacing the sulfur atom with selenium reduces the vibrational frequencies of the optical modes, causing less separation between the branches, reflecting the greater atomic mass of selenium. However, for MoWS_2_Se_2_ (Figure 12c), the dispersion curves are intermediate in relation to the previous two, showing a mixture of vibrational modes, reflecting the flattening of some optical branches. Thus, given these results, dynamic stability is observed in all alloys, showing a clear tendency for optical mode frequencies to decrease when S is replaced by Se, with a bandgap between acoustic and optical modes possibly favoring applications in thermoelectronic and optoelectronic devices due to the reduction of phonon–phonon scattering.

### 3.6Infrared and Raman Spectra

Employing the GGA-PBE-optimized structures, Density Functional Perturbation Theory (DFPT)^103^ calculations are performed to analytically determine the second derivative of the total energy concerning perturbations that include atomic displacements, electric fields, and variations in crystalline lattice parameters.^104^ Figure 13 shows the infrared and Raman spectra for MoWS_4_ (black lines), MoWSe_4_ (dashed red lines), and MoWS_2_Se_2_ (dashed blue lines), where all positive frequencies confirm that these structures represent local energy minima.

**Figure 13:**
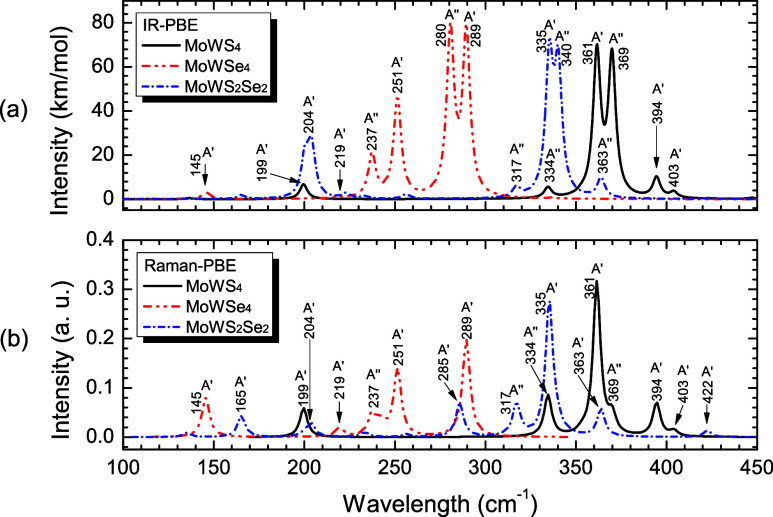
(a) Infrared and (b) Raman spectrum of MoWS_4_ (solid black lines), MoWSe_4_ (dashed red lines), and MoWS_2_Se_2_ (dotted blue lines) in the 100–450 cm^–1^ range, with their respective normal modes (for interpretation of the references to colors in this figure legend, the reader is referred to the web version of this paper).

The 2D TMD-MoWX_4_ (X = S, Se) monolayers and Janus TMD-MoWS_2_Se_2_ possess 6 atoms per unit cell, with each atom contributing 3 degrees of freedom (*x*, *y*, *z* displacements), yielding 18 active modes (6 × 3 = 18) at the high-symmetry Γ point in the Brillouin Zone. Among these, 3 correspond to acoustic modes (system translations), while the remaining 15 are optical modes (internal lattice vibrations). The *P*_1_ *m*_1_ space group is noncentrosymmetric, featuring only a mirror plane (*m*) perpendicular to the *b* (*y*)-axis while lacking inversion symmetry. This absence of centrosymmetry allows all vibrational modes to be simultaneously active in both IR (infrared) and Raman spectroscopy, as the mutual exclusion between IR and Raman modes—typical of centrosymmetric systems—does not apply.

In *P*_1_ *m*_1_, each normal vibrational mode can couple with both the electric dipole moment (IR-active) and the polarizability (Raman-active), enabling a more comprehensive vibrational characterization in noncentrosymmetric crystals. In such systems, the polar structure and concurrent spectroscopic activity are directly linked to the material’s physical properties.^105^

In the infrared spectra (Figure 13a), the most intense peaks that appear are as follow: at 361.51 cm^–1^ (361) and 369.88 cm^–1^ (369) for MoWS_4_ (376.5 cm^–1^ for MoS_2_ and 355.8 cm^–1^ for WS_2_),^106^ corresponding to an out-of-plane bending (wagging) movement of S–W–S and S–Mo–S, parallel to the *x*- and *y*-axis, with irreducible representations *A*′ and *A*″, respectively; at 280.68 cm^–1^ (280) and 289.44 cm^–1^ (289) for MoWSe_4_ (283.7 cm^–1^ for MoSe_2_ and 249.7 cm^–1^ for WSe_2_),^106^ assigned mainly to an out-of-plane bending (wagging) movement of S–Mo–S parallel to the *y*- and *x*-axis with irreducible representations *A*″ and *A*′, respectively; and 335.26 cm^–1^ (335) and 340.19 cm^–1^ (340) for MoWS_2_Se_2_, related mainly to a S–W–S rocking movement along the *x*-axis, and to a S–Mo–Se rocking movement along the *y*-axis, with irreducible representations *A*′ and *A*″, respectively. The Raman spectra (Figure 13b) feature, for MoWS_4_, a dominant peak at 361 cm^–1^ (*A*′ mode) with secondary modes at 199 cm^–1^ (*A*′ mode), 334 cm^–1^ (*A*″ mode), 369 cm^–1^ (*A*″ mode), and 394 cm^–1^ (*A*′ mode) (376.5 and 392.4 cm^–1^ for MoS_2_; 355.8 and 415.4 cm^–1^ for WS_2_),^106^ while for MoWSe_4_, a prominent peak is featured at 289 cm^–1^ (*A*′ mode) followed by 251 cm^–1^ (*A*′ mode) (283.7 and 238.4 cm^–1^ for MoSe_2_; 249.7 and 249.9 cm^–1^ for WSe_2_),^106^ and last for MoWS_2_Se_2_, the most intense peak is featured at 335 cm^–1^ (*A*′ mode), accompanied by modes at 285 cm^–1^ (*A*′ mode) and 317 cm^–1^ (*A*″ mode).

### 3.7Thermodynamic Properties

Figure 14a shows the thermodynamic potential curves, including enthalpy (*H*), T × entropy (T × S), and free energy (*F*), as a function of temperature (T) for the two-dimensional MoWSSe (MoWS_4_, MoWSe_4_, and MoWS_2_Se_2_). The enthalpy curves (solid, dashed, and dotted black lines) exhibit nearly linear behavior between 0 and 1000 K, with little discrepancy between the values of the different MoWSSe monolayer configurations. The phonon free energy (solid, dashed, and dotted red lines) is positive at 0 K due to zero-point vibrations, decreases slightly to about 600 K, and then assumes linear behavior as the temperature increases. The zero-point energies are 0.312, 0.214, and 0.261 eV for MoWS_4_, MoWSe_4_, and MoWS_2_Se_2_, respectively. The T × S term (solid, dashed, and dotted blue lines) increases exponentially with temperature, increasing the atomic vibration of the monolayer, with energy values of MoWSe_4_ > MoWS_2_Se_2_ > MoWS_4_ above 250 K.

**Figure 14:**
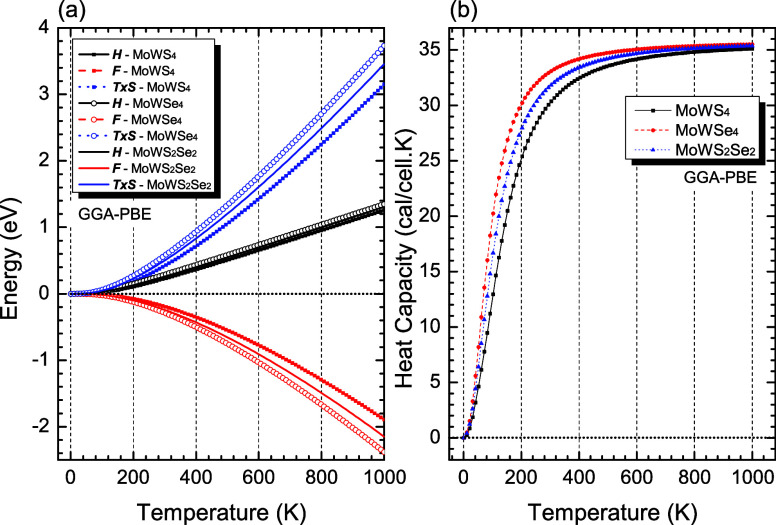
(a) Thermodynamic properties for the MoWS_4_, MoWSe_4_, and MoWS_2_Se_2_ monolayers: enthalpy (*H*), free energy (*F*), and temperature × entropy (*T* × *S*). (b) Constant volume heat capacity *C*_V_ for the MoWS_4_ (black squares), MoWSe_4_ (red circles), and MoWS_2_Se_2_ (blue triangles) monolayers as a function of the temperature (for interpretation of the references to colors in this figure legend, the reader is referred to the web version of this paper).

Figure 14b shows the constant volume heat capacity (*C*_V_) as a function of temperature for MoWS_4_, MoWSe_4_, and MoWS_2_Se_2_, revealing that the heat capacity increases rapidly from 0 to 600 K. MoWS_4_, MoWSe_4_, and MoWS_2_Se_2_ heat capacity diverge in value from slightly above 0 to 600 K, where the values begin to saturate and overlap, with MoWSe_4_ having the highest heat capacity among the three in this interval and MoWS_4_ having the lowest heat capacity.

### 3.8Molecular Quantum Dynamics

The thermal stability of the MoWS_4_, MoWSe_4_, and MoWS_2_Se_2_ structures was investigated by unpolarized quantum dynamics using LDA-PWC^73–75^ and DNP basis sets through the DMol3 software.^68,69^ Ab initio dynamics simulations were based on the Nosé-Hoover thermostat^70–72^ for a supercell 2 × 2 × 1 with a time step of 1 fs to ensure the stability of TMD and TMD Janus alloys at a temperature of 300 K. The cells maintained their structure, and no chemical bond rupture was observed, showing minor deformations in all monolayers of the TMD alloy. Figure 15 shows the behavior of the potential energy (in Ha) as a function of time (in picoseconds), in which there are some thermal fluctuations. However, as time passes during the dynamics, the fluctuations decrease, indicating the reliability of the simulation.

**Figure 15:**
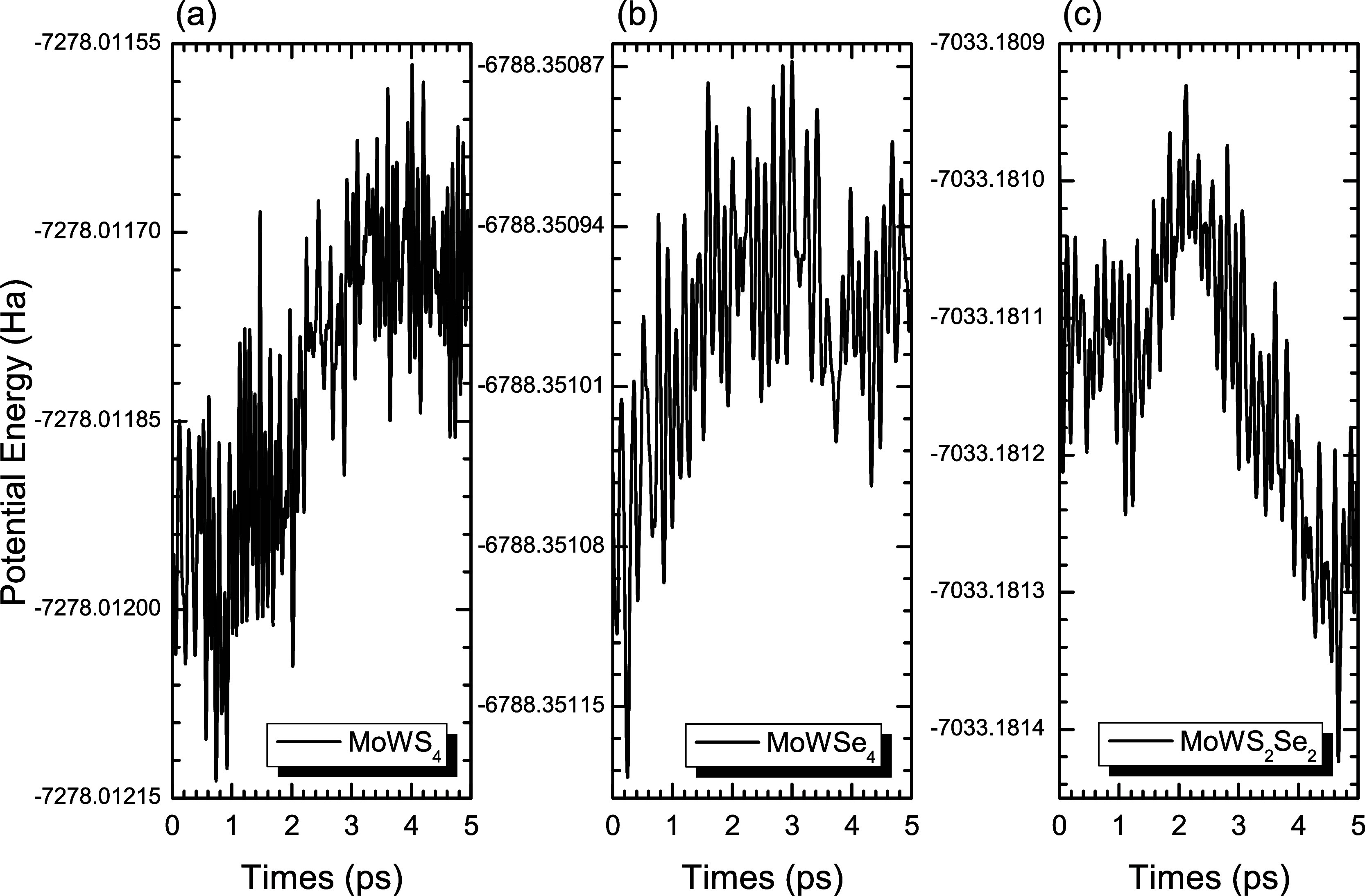
Potential energy (Ha) × time (ps) graphic for (a) MoWS_4_, (b) MoWSe_4_, and (c) MoWS_2_Se_2_ monolayers in quantum dynamics simulation considering the *NVT* ensemble.

## 4Conclusions

This study presents a comprehensive investigation of the structural, electronic, optical, and vibrational properties of MoWS_4_, MoWSe_4_, and Janus MoWS_2_Se_2_ monolayers using Density Functional Theory (DFT) and Density Functional Perturbation Theory (DFPT) calculations. The results demonstrated that all structures are dynamically stable, as evidenced by the absence of imaginary phonon modes in the dispersion curves, with 18 vibrational modes identified (3 acoustic and 15 optical). The cohesive energy shows that all of the TMD alloys presented energetic stability. Furthermore, the formation energies indicate that all monolayers are thermodynamically stable. Electronic structure analysis revealed direct bandgaps along the Γ – *Y* path, with significantly larger values calculated using the HSE06 functional (2.21, 1.95, and 2.08 eV) compared to GGA-PBE (1.68, 1.47, and 1.58 eV), highlighting the importance of advanced exchange correlation methods for accurate predictions. The PBE + SOC calculations also predict direct bandgaps of 1.53 eV (MoWS_4_), 1.33 eV (MoWSe_4_), and 1.43 eV (MoWS_2_Se_2_), respectively. Furthermore, SOC reveals band splitting at the Γ → *Y* high-symmetry point within the conduction and valence states in all three studied monolayer structures. The projected density of states (PDOS) showed the dominance of Mo and W d-orbitals near the Fermi level, while the contribution of chalcogen (S/Se) p-orbitals directly influenced the optical properties.

The dielectric functions and optical absorption spectra exhibited distinct behaviors among the materials, with MoWS_4_ and MoWS_2_Se_2_ showing strong absorption peaks around 10 eV, while MoWSe_4_ displayed a shift to lower energies (9.48 eV), attributed to selenium’s larger atomic radius and higher polarizability. Optical anisotropy was moderate, with more pronounced variations along the [001] direction and in polycrystalline samples. Furthermore, infrared and Raman spectra revealed unique vibrational signatures for each composition, with modes simultaneously active in both spectra due to the noncentrosymmetric *P*_1_ *m*_1_ symmetry. The most intense IR peaks appeared at 361 cm^–1^ (MoWS_4_), 280 cm^–1^ (MoWSe_4_), and 335 cm^–1^ (MoWS_2_Se_2_), while the dominant Raman modes were observed at 361 cm^–1^ (MoWS_4_), 289 cm^–1^ (MoWSe_4_), and 335 cm^–1^ (MoWS_2_Se_2_), providing valuable tools for experimental characterization.

From a thermodynamic perspective, the negative free energies at all temperatures (0 to 1000 K) confirmed the dynamical stability of each monolayer, with MoWSe_4_ exhibiting the highest thermodynamic stability, followed by MoWS_2_Se_2_ and MoWS_4_. The heat capacity (*C*_v_) increased rapidly up to 600 K, saturating at higher temperatures, with MoWSe_4_ showing the highest values, consistent with its higher vibrational density of states. The small fluctuations in quantum dynamics are natural, indicating thermal oscillations, but each alloy showed a stable potential energy curve without large variations, confirming good thermal stability. These results indicate the potential of these materials for applications in optoelectronic devices, thermoelectrics, and sensors where precise control of electronic and vibrational properties is essential. Future studies could explore the effects of mechanical strain, doping, and experimental synthesis of these monolayers to validate theoretical predictions and optimize their performance for technological applications. In summary, this work provides a solid foundation for the rational design of MoWX_4_-based 2D materials, integrating multiple physical properties into a unified theoretical framework.

## Data Availability

All data are available in this manuscript.
